# Hyperhomocysteinemia Accelerates Collagen Accumulation in the Adventitia of Balloon-Injured Rat Carotid Arteries via Angiotensin II Type 1 Receptor

**DOI:** 10.3390/ijms151119487

**Published:** 2014-10-27

**Authors:** Dan Yao, Ning-Ling Sun

**Affiliations:** Department of Cardiology, Peking University People’s Hospital, No. 11 Xizhimen South Street, Xicheng District of Beijing, Beijing 100044, China; E-Mail: yaodan-716@163.com

**Keywords:** hyperhomocysteinemia, homocysteine, adverntitia, collagen, Ang II type 1 receptor

## Abstract

Recent studies suggest that hyperhomocysteinemia (HHcy) increases collagen type I accumulation in rat vascular adventitia after balloon injury and that Angiotensin II (Ang II) induces collagen synthesis in vascular adventitial fibroblasts. Reports also indicate that Ang II type1 receptor (AT_1_R) activation, mediated by homocysteine (Hcy) may contribute to collagen type 1 expression in mouse aortic endothelial cells. However, little is known about the possible mechanisms behind the relationship between Hcy and AT_1_R in adventitial remodeling. Thus, we investigated whether HHcy induces collagen accumulation via activation of AT_1_R in the adventitia. Male Sprague-Dawley (SD) rats were randomly divided into a control group and a 1% l-methionine-induced HHcy group. Balloon injury was performed after 12 experimental weeks and animals were sacrificed at 7, 14, and 28 days after injury. Collagen deposition and AT_1_R expression was measured with Western blot. Serum Hcy, adventitial collagen, and AT_1_R levels were higher in the HHcy group compared with the control group. Hcy time-dependently induced collagen type 1 and AT_1_R expression, with the highest induction observed at 48 h. Also, we observed that the AT_1_R blocker, valsartan, attenuated collagen type 1 and AT_1_R expression. HHcy exacerbates adventitial remodeling after balloon injury, and the underling mechanisms may be related to AT_1_R activity.

## 1. Introduction

Vascular remodeling is defined as structural changes in the vascular wall due to cell and extracellular matrix dysfunction [1]. Although all three layers of the arterial wall (intima, media, and adventitia) contribute to vascular remodeling, they each have their own unique structures and properties, and therefore display varied responses to arterial wall injuries. Most investigations have focused on the intima in injury-induced arterial remodeling. However, recent experimental data suggest that adventitial fibroblasts are important initial steps in vascular remodeling after injury [2]. Collagen type 1 is a major structural protein in normal and diseased arterial walls. Fibrosis caused by excessive deposition of collagen is a structural hallmark of vascular disease and contributes to increased vascular stiffness [3,4]. One of the sources of collagen secreting cells is differentiated adventitial fibroblasts.

Homocysteine (Hcy) is a sulfur-containing amino acid and high concentrations of Hcy are usually caused by genetic disorders, kidney disease, a high methionine diet, or vitamin deficiencies [5]. Moderate hyperhomocysteinemia (HHcy) is considered an independent risk factor of stroke, myocardial infarction, and atherosclerosis [6,7,8]. Clinical studies reveal a positive association between HHcy and the risk of restenosis after percutaneous angioplasty [9,10,11]. Moreover, animal studies indicate that HHcy exacerbates neointimal formation and adventitial collagen accumulation in the balloon-injured rat [12,13]. In addition, data from * in vitro* experiments show that HHcy increases collagen type 1 expression in cultured mouse aortic endothelial cells (MAECs) [14] and rat aortic adventitial fibroblasts [12].

Ang II type 1 receptor (AT_1_R) is a key plasma membrane receptor of the renin-angiotensin system. AT_1_R activation leads to cardiac remodeling, ventricular hypertrophy, neointima formation, and smooth muscle cell proliferation and migration [15,16]. Although recent studies indicate that HHcy induced activation of AT_1_R regulates collagen type 1 expression in mouse aortic endothelial cells [14], little is known about the association between HHcy and AT_1_R in adventitial remodeling. Therefore, we investigated whether HHcy stimulates adventitial collagen type 1 expression after a balloon-injury procedure in the rat via AT_1_R activation.

## 2. Results and Discussion

### 2.1. Serum Total Hcy

Serum total Hcy was significantly higher in the HHcy group (26.6 ± 1.1) μmol/L compared with the control group ((4.9 ± 0.1) μmol/L, *p* < 0.01) after a 1% l-methionine diet for 12 weeks.

### 2.2. Effect of HHcy on Neointima Formation and Adventitial Hyperplasia

**Figure 1:**
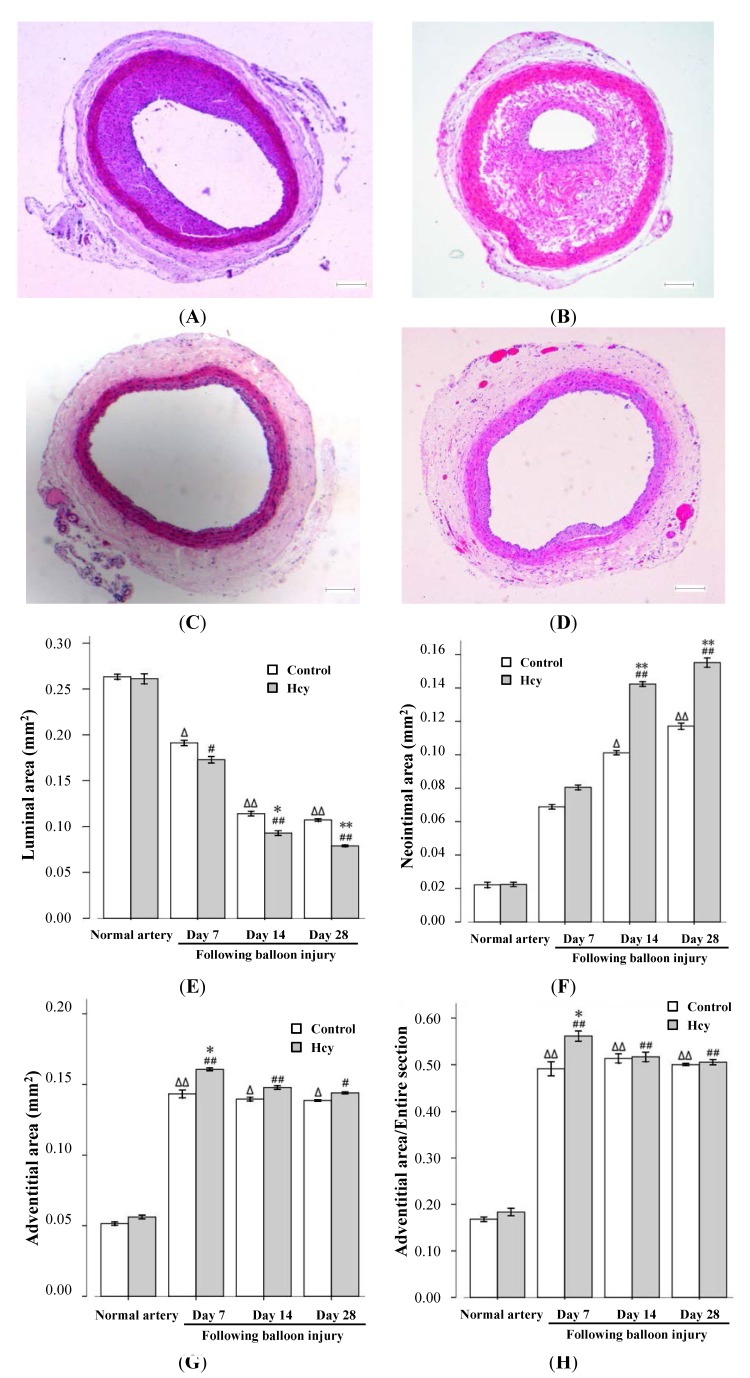
Effect of homocysteine on neointima formation and adventitial hyperplasia of balloon injured arteries. (HE, hematoxylin and eosin stain ×50).The neointimal areas in control group were smaller than that in hyperhomocysteinemia (HHcy) group at day 28 after balloon injury ((**A**) control group, (**B**) HHcy group); Adventitial hyperplasia in control group was observed at 7 days of balloon injury (**C**); Adventitial hyperplasia in HHcy group futher increased at the same time point (**D**); and (**E**–**H**)Effect of homocysteine on luminal area, neointimalarea adventitial changes in the normal artery (non-injured carotid artery) and injured arteries. Scale bars = 100 μm. ^Δ^ *p* < 0.05, ^ΔΔ^ *p* < 0.01 *versus* normal artery with control diet; ^#^ *p* < 0.05, ^##^ *p* < 0.01 *versus* normal artery with l-methionine-induced HHcy group; * *p* < 0.05, ** *p* < 0.01 *versus* control group of the same time point.

HHcy markedly reduced the luminal area, increased neointimal thickness and induced adventitial hyperplasia after injury (Figure 1). Compared with intact arteries, the luminal areas of injured arteries in both the control and the HHcy group were significantly smaller at day 28 after injury, and these areas were significantly smaller in the HHcy group compared with the control group at both day 14 and 28 after balloon injury. In contrast, the neointimal area was significantly larger in the HHcy group compared to controls at day 14 and 28 after balloon injury.

The adventitial area in the HHcy group was larger than in controls. Differences existed mainly at day 7 after injury in the two groups ((0.14 ± 0.01) *versus* (0.16 ± 0.01) mm^2^, *p* < 0.05).

### 2.3. Effect of HHcy on Adventitial Expression of Collagen Type 1 and AT_1_R in Balloon-Injured Arteries

**Figure 2:**
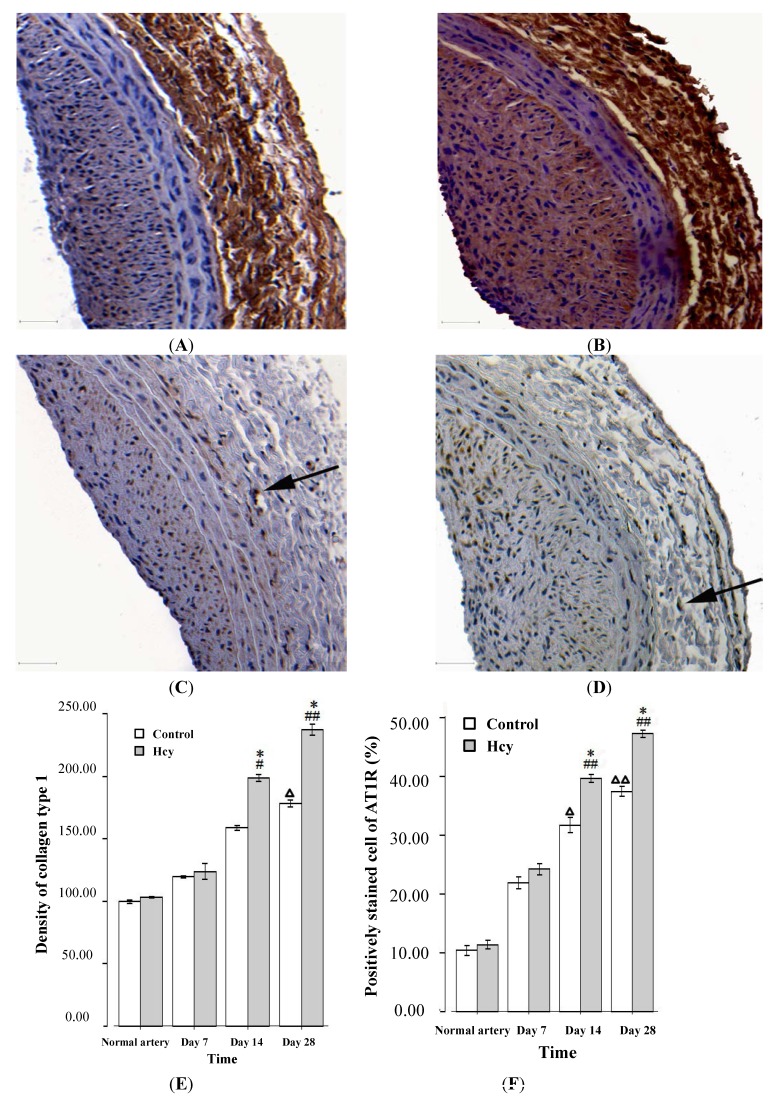
Effect of homocysteine on the expression of collagen type 1 (**A**,**B**) and AT_1_R (**C**,**D**) in adventitia of balloon-injured arteries; scale bars = 50 μm. Immunohistochemical staining shows the expression of collagen type 1 and AT_1_R in both control group and HHcy group at day 28 following injury ((**A**,**C**) the control group; (**B**,**D**) the HHcy group ×200)); (**E**) Optical density of adventitial collagen type 1 staining in each group at different time points; and (**F**) Percentage of AT_1_R positively stained cell in the adventitia. ^Δ^ *p* < 0.05, ^ΔΔ^ *p* < 0.01 *versus* normal artery with control diet; ^#^ *p* < 0.05, ^##^ *p* < 0.01 *versus* normal artery with methionine diet; * *p* < 0.05 *versus* control group of the same time point.

As shown in Figure 2A–D, adventitial deposition of collagen type 1 and expression of AT_1_R increased at day 28 after balloon injury. Data from immunohistochemical studies indicates that (Figure 2E,F) the mean optical density of collagen type 1 staining and the percentage of AT_1_R positive cells in the HHcy group were significantly increased compared with controls at day 14 and 28 after balloon injury.

### 2.4. Effect of l-Hcy on Collagen Type 1 and AT1R Expression in Cultured Adventitial Fibroblasts

**Figure 3:**
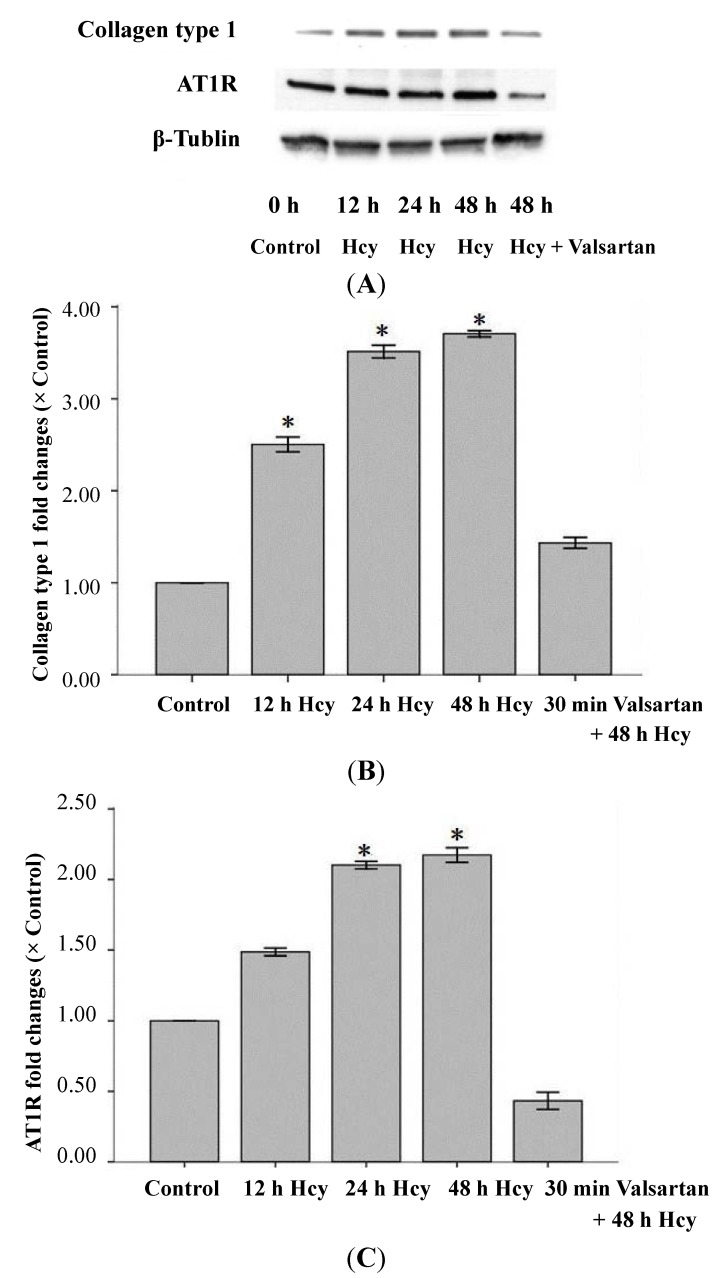
Effect of homocysteine on the expression of collagen type 1 and AT_1_R in cultured adventitial fibroblasts. (**A**) Western blot indicated collagen type 1 and AT_1_R levels in cells treated with l-Hcy(100 µmol/L) for different periods of time, or pretreated with valsartan (100 nmol/L) for 30 min followed by l-Hcy(100 µmol/L) treatment for 48 h (*n* = 4).Densitometric analyses of collagen type 1 and AT_1_R were performed against β-tubulin. * *p* < 0.05 compared with controls (**B**,**C**).

l-Hcy (Sigma, St. Louis, MO, USA) induced collagen type 1 and AT_1_R expression in a time-dependent manner as indicated by using Western blot (Figure 3). There was a 2~3-fold increase in collagen type 1 and AT_1_R expression at 48 h. The AT_1_R inhibitor valsartan markedly attenuated collagen type 1 and AT_1_R expression.

### 2.5. Discussion

HHcy is widely recognized as an independent risk factor responsible for restenosis after percutaneous angioplasty and for increased aortic stiffness in the general population [17,18]. HHcy’s effect on intimal hyperplasia has also been documented in balloon-injured rat carotid arteries, but recent studies have focused on the outermost adventitial layer rather than on the innermost layer. After injury, adventitial fibroblasts are activated and differentiated to myofibroblasts, which secrete extracellular matrix proteins, such as collagen [19]. HHcy exacerbates vascular constrictive remodeling by accelerating neointima formation and collagen accumulation in the adventitia [9]. Our data are consistent with the above mentioned studies, but we further demonstrated an increase in AT_1_R expression after balloon injury in HHcy rat carotid vascular adventitia.

We demonstrated that incubation of rat aortic adventitial fibroblasts with l-Hcy significantly increased collagen type 1 and AT_1_R expression, suggesting that adventitial fibroblasts may play an important role in the accumulation of the extracellular matrix and vascular adventitial remodeling. Also, the AT_1_R inhibitor, valsartan, administered 30 min before Hcy treatment markedly attenuated both collagen type 1 and AT_1_R expression. Our results suggest that HHcy activates AT_1_R, thereby regulating collagen type 1 expression of in adventitial fibroblasts.

Adventitial fibroblasts are critical to the adventitial response to arterial wall injury, differentiating into myofibroblasts-migrating, proliferating, and secreting procollagen-1, which exacerbates perivascular fibrosis and vascular remodeling [20]. Previous studies suggest a positive relationship between collagen content and restenosis. HHcy, an independent risk factor for restenosis, increases collagen accumulation in cultured aortic endothelial cells [11] and vascular fibroblasts [9]. In line with these reports, our work suggests that HHcy exacerbates collagen deposition in the adventitia. However, in this study we did not use AT_1_R antagonist in animal model. Further studies dealing with this point and mechanism about how Hcy interacts with AT_1_R.

Ang II, the main effector hormone of the renin-angiotensin system, has been shown to modulate cell migration, rates of growth and apoptosis, and the extent of extracellular matrix deposition leading to ventricular hypertrophy and atherothrombosis through its receptors [21,22]. However, there is limited information on the possible relationship between Hcy and Ang II in vascular remodeling. Ang II plays a central role in the pathophysiology of cardiovascular through AT_1_R. Recently, experiments have indicated that HHcy aggravated Ang II-induced abdominal aortic aneurysm, which strongly suggests that Hcy may promote aortic aneurysm via AT_1_R, at least in part by adventitial activation. Valsartan, a selective AT_1_R antagonist, attenuates pathological ventricular hypertrophy induced by hyperhomocysteinemia in rat [16] and decreases HHcy-mediated collagen type 1 formation in endothelial cells [11]. Therefore, we assessed the expression of collagen and AT_1_R in cultured adventitial fibroblasts after incubation with l-Hcy and found that valsartan significantly reduced collagen deposition in cultured vascular fibroblasts, indicating that the potential promising benefit of AT_1_R for patients with HHcy.

## 3. Experimental Section

### 3.1. Animals and Treatments

The study was approved by the Department of Laboratory Animal Science of Peking University Health Science Center and performed in accordance with the guidelines for care and use of laboratory animals. Forty-eight Male SD rats weighing 150–200 g were purchased from Peking University Health Science Center (Beijing, China) and housed two per cage in an air-conditioned room at (22 ± 1) °C under a 12 h dark-light cycle. The animals were divided into two groups: control group with a normal rat chow diet (*n* = 24) and a HHcy group, which was given a 1% l-methionine diet (*n* = 24) [13]. Animals were fed for 12 weeks prior to balloon injury and were continually fed their group diet until the end of each experiment.

### 3.2. Measurement of Serum Homocysteine Level

Blood was collected from the posterior orbital venous plexus from all the rats after overnight fasting to determine the levels of serum Hcy. Total serum Hcy level was quantified by enzymatic cycling assay [23,24].

### 3.3. Balloon Injury of Rat Carotid Artery

Rats were anesthetized with 10% chloral hydrate (300 mg/kg, intraperitoneal injection (ip.)). The left common, external and internal carotid arteries were exposed through a longitudinal midline cervical incision. A 2F Fogarty catheter (Cordis, Miami, FL, USA) was introduced into the external carotid artery via an arteriotomy, inflated with normal saline, and withdrawn 3 times with rotation to denude the left common carotid arteries. After removal of the catheter, the external carotid was ligated and the clamped common carotid artery was opened so that blood flow could resume [25]. Normal artery had only the operative procedure, but no balloon insertion.

### 3.4. Histology and Morphometry

At the end of the experiment, all rats were euthanized with chloral hydrate. The injured left common carotid artery and the non-injured artery were collected, fixed in 10% neutral buffered formalin and embeded in paraffin. Each paraffin block were cut into 5 µm sections and stained with hematoxylin and eosin. The luminal, neointimal, and adventitial areas were inspected for collagen type I and AT_1_R expression using Image Pro Plus 6.0 software (Media Cybernetics, Sliver Spring, MD, USA).

### 3.5. Immunohistochemistry

The expression of collagen type 1 and AT_1_R in the adventitia were assessed by immunohistochemistry staining. Deparaffinized and rehydrated 4 µm sections of left common carotid arteries were incubated in 10% normal goat serum for 30 min at room temperature, and then they were incubated with rabbit anti-rat collagen type I (1:200; Abcam, Cambridge, UK) and AT_1_R (1:100; Abcam) at 4 °C overnight. The sections were then incubated with biotinylated goat anti-rabbit IgG for 30 min, washed and incubated in diaminobenzidine (DAB) chromogen until a color change was observed under the microscope. Negative controls (omission of primary antibody) were prepared as described previously [26]. Then, three immunostained sections per artery were visualized microscopically (×200). In four fields of each section, collagen content was assessed by mean optical density and AT_1_R expression was measured by counting the number of positively and negatively stained cells and calculating the percentage of positively stained cells in total cells.

### 3.6. Cell Culture of Vascular Fibroblasts

Adventitial aortal fibroblasts were prepared according to the method of Kim and colleagues [27] and Tsuruda’s group [28] with some modifications. Rats were anesthetized with chloral hydrate and rapidly decapitated. Thoracic aorta were isolated from 8 week-old male SD rats and placed in Dulbecco’s modified Eagle’s medium (DMEM, Gibco, Gaithersburg, MD, USA) containing 10% fetal bovine serum (Gibco) and antibiotics. The adventitia was peeled from the underling media and cut into 1 mm^2^ segments under sterile conditions. Fibroblasts were grown until large cell colonies formed from surrounding explants in approximately 4–7 days. After achieving confluence, cells were harvested with trypsin and used for experiments at passage 3–5. For all experiments, cells were grown to 70%–80% confluence and then cells were made quiescent by incubation in DMEM supplemented with 0.1% fetal serum albumin (FSA, Gibco) for 24 h before stimulation. Adventitial fibroblasts were treated with l-Hcy (100 µmol/L, Sigma, St. Louis, MO, USA) for 0, 12, 24, and 48 h to measure collagen type 1 and AT_1_R expression. Simultaneously, cells were pretreated with valsartan (100 nmol/L, Sigma) for 30 min followed by treatment with Hcy (100 µmol/L, Sigma) for 48 h.

### 3.7. Western Blot

Protein was extracted from cultured adventitial fibroblasts, separated with 8% SDS-PAGE and then transferred onto polyvinylidene difluoride membrane (Bio-Rad, Hercules, CA, USA). Membranes were blocked with 5% non-fat milk for 1 h at room temperature and were incubated with anti-collagen type 1 antibody (1:5000; Abcam), anti-AT_1_R (1:500; Abcam), β-tubulin (1:10,000; Epitomics, Burlingame, CA, USA) followed by incubation with horseradish peroxidase-coupled secondary antibody. Immunoreactive bands were visualized with enhanced chemiluminescence (ECL) and optical density was measured with Image Lab software (3.0 Bio-Rad, Hercules, CA, USA).

### 3.8. Statistical Analysis

The data are expressed as means ± standard error (SE). Statistical analysis was carried out with SPSS version 13.0 (SPSS Inc., Chicago, IL, USA). Comparisons between groups were made using the Student’s *t*-test and one-way ANOVA followed by a Bonferroni’s test for parameters with normal distribution. Statistical significance was accepted at *p* < 0.05.

## 4. Conclusions

HHcy exacerbated vascular remodeling by increased collagen accumulation and AT_1_R expression in the adventitia of balloon-injured rat. Moreover, AT_1_R antagonist, valsartan attenuated l-Hcy induced collagen type 1 expression and AT_1_R expression in cultured adventitial fibroblasts. Although the detailed mechanism between HHcy and AT_1_R in vascular remodeling is still incompletely defined, our study suggest that AT_1_R antagonist may become a beneficial drug for HHcy patients to prevent vascular reverse remodeling and restenosis after angioplasty.
